# Comparative Transcriptomic Analysis Reveals the Regulated Expression Profiles in *Oreochromis niloticus* in Response to Coinfection of *Streptococcus agalactiae* and *Streptococcus iniae*

**DOI:** 10.3389/fgene.2022.782957

**Published:** 2022-03-03

**Authors:** Miao Cui, Zibin Wang, Yongchun Yang, Ru Liu, Min Wu, Yujie Li, Qizhong Zhang, Delin Xu

**Affiliations:** College of Life Science and Technology, Jinan University, Guangzhou, China

**Keywords:** tilapia, transcriptomics, *Streptococcus agalactiae*, *Streptococcus iniae*, immune responses, intestine

## Abstract

Tilapia (*Oreochromis* sp.) is one of the important economical fishes in the world. Streptococcosis is commonly found in tilapia, causing severe and devastating effects in tilapia cultures. *Streptococcus agalactiae* and *Streptococcus iniae* are the predominant pathogens causing tilapia streptococcosis. To understand the molecular mechanisms underlying differential streptococcal infection patterns, Nile tilapias (*Oreochromis niloticus*) were infected by 1 × 10^7^ CFU/mL *S. agalactiae*, 1 × 10^7^ CFU/mL *S. iniae*, and 1 × 10^7^ CFU/mL *S. agalactiae* and *S. iniae* (1:1), respectively, and transcriptome analysis was conducted to the intestine samples of Nile tilapia (*Oreochromis niloticus*) at 6, 12, 24 h, and 7 days post-infection. A total of 6,185 genes that differentially expressed among groups were identified. Eight differentially expressed genes (DEGs) including *E3 ubiquitin-protein ligase TRIM39-like*, *C-X-C motif chemokine 10-like(CXCL 10)*, *C-C motif chemokine 19-like*, *interleukin-1 beta-like*, *IgM heavy chain VH region*, *partial*, *IgG Fc-binding protein*, *proteasome subunit beta type-8* (*PSMB8*), and *ATP synthase F(0) complex subunit B1*, *mitochondrial* that involved in the immune system were selected, and their expression levels in the coinfection group were significantly higher than those in either of the single infection groups. These genes were associated with four different KEGG pathways. Additionally, the differential expression of eight DEGs was validated by using the RT-qPCR approach, and their immunological importance was discussed. The results provided insights into the responses of tilapia against *S. agalactiae* and *S. iniae* at the transcriptome level, promoting our better understanding of immune responses for aquatic animal against *Streptococcus*.

## 1 Introduction

Tilapia (*Oreochromis niloticus*) is an economic and important aquaculture fish species in the world. China is the world’s largest producer of tilapia. In 2020, the aquaculture production of tilapia reached 1.6417 million tons, accounting for about 40% of the world’s total tilapia production, and the export volume of tilapia ranked among the top three in aquatic product exports in China (Yearbook, 2020). However, with the rapid development of the tilapia aquaculture industry, tilapia cultures have encountered tremendous challenges caused by disease outbreaks. As a common disease caused by pathogenic bacteria, streptococcosis has devastating effects in tilapia cultures (Anshary et al., 2014). Gram-positive bacteria *S. agalactiae* and *S. iniae* are the main pathogens of streptococcosis (Maulu et al., 2021). In 2019, the economic losses for the tilapia aquaculture industry caused by *S. agalactiae* and *S. iniae* have reached around 2.5 billion RMB (Huang et al., 2014; Tavares et al., 2018; Shiry et al., 2019). *S. agalactiae* is also known as group B *Streptococcus* (Brown, 1939). Both of them are recognized as causative agents of zoonosis with a broad host range, including horse, pig, and fish (Mohan, 1947; Monteverde and Simeone, 1951; Poppert et al., 2009). Typical symptoms of the *Streptococcus*-infected tilapia include blackening of the fish surface, prominent or turbid eyeballs, whiteness, bleeding, abdominal spots, and bleeding on the inner side of the lid (Perera et al., 1994). Previous research studies on tilapia infected by *S. agalactiae* and *S. iniae* focused mainly on the isolation, identification and typing of strains, screening of drugs for the prevention and treatment of *Streptococcus*-infected tilapia, the drug resistance, and vaccine development (Suanyuk et al., 2008; Cai et al., 2020; De Sousa et al., 2021). Most research studies on the immune mechanism of tilapia mainly focused on the kidney and spleen (Zhu et al., 2015; Zhu et al., 2017). However, the knowledge of the molecular mechanisms in immune response aspects against *S. agalactiae* and *S. iniae* in the intestine of tilapia is still limited.

The outbreak of the disease is often caused by the joint action of multiple pathogens (Austin and Allen-Austin, 1985). For example, *S. agalactiae* and *S. iniae* have previous been isolated from tilapia suffering from streptococcosis (Chen et al., 2007), indicating that coinfection of the two pathogens is common in tilapia. Although the transcriptome profiling in the spleen or kidney of tilapia (*Oreochromis niloticus*) infected by *Streptococcus agalactiae* at the early stage has been studied previously (Zhang et al., 2013), investigations regarding the effect caused by coinfection are still lacking (Li et al., 2014).

With the rapid development of high-throughput sequencing, transcriptome analysis has been widely applied to investigate molecular mechanisms underlying host immune response upon infections. For example, Wang et al. carried out transcriptome sequencing of spleen samples from *Oncorhynchus mykiss* infected with *Yersinia ruckeri* and identified many KEGG pathways and differentially expressed genes associated with the immune system including *CCR9*, *CXCL11*, *IL-1 beta*, and *CARD9* (Wang et al., 2021). Zhang et al. identified various essential genes which play roles on regulating myogenesis from the transcriptome analysis of *Trichiurus lepturus* (Zhang et al., 2016). Tilapia is a freshwater teleost species, and the defense action against infection is mainly achieved by the innate immune system (Kordon et al., 2018). The mucosal immune system in the intestine of tilapia is an important barrier to infectious microorganisms (Andani et al., 2012). In spite of classical immune organs, a recent study revealed that the intestinal epithelium is the main entry site of *Streptococcus* in tilapia, and the intestine plays an important defensive role against *Streptococcus* (Iregui et al., 2016). The impact of *Streptococcus* on the intestine of tilapia warrants further study to better understand the role of intestinal immune function in bony fish against *Streptococcus*. In this study, transcriptome analysis was conducted in tilapia challenged by coinfections of *S. agalactiae* and *S. iniae*, as well as by single infections of each of the two pathogens. Differentially expressed genes (DEG) and their corresponding metabolic pathways in the intestine were identified. The molecular mechanisms underlying the immune responses of tilapia following infections are discussed.

## 2 Materials and Methods

### 2.1 Bacterial Strains and Culture Condition

The *S. agalactiae* standard strain ATCC13813 and the *S. iniae* standard strain ATCC29178 were donated by the Pearl River Fisheries Research Institute of Chinese Academy of Fishery Sciences (Shin et al., 2006; Guo et al., 2017). The bacteria were identified and grown in BHI liquid shaken cultures (180 rpm and 28°C) for one day.

### 2.2 Nile Tilapia Collection, Maintenance, and Treatment

*O. niloticus* (24 ± 1.02 g) were obtained from the Guangdong Tilapia Fine Breeding Farm (each fish was free from *Streptococcus* spp.). Before experimental challenge, the fish were acclimated in the laboratory (28°C, 6.8–7.2 pH) for 2 weeks. Tilapia were randomly divided into four groups, including the single infection of *S. agalactiae* (AG), the single infection of *S. iniae* (IG), and the coinfection of *S. agalactiae* and *S. iniae* (MG) and control (CG), with 60 fish in each tank at a temperature of 28 ± 0.5°C. The fish in the AG, IG, and MG groups were injected with 0.3 ml final concentration of 1 × 10^7^ CFU/ml *S. agalactiae*, 1 × 10^7^ CFU/ml *S. iniae*, and 1 × 10^7^ CFU/mL *S. agalactiae* and *S. iniae* (1:1), respectively (lethal concentration 50%). Meanwhile, the fish in the control group were injected with 0.3 ml normal saline and cultured in a tank with a continuous supply of water. At 0, 6, 12, 24 h, and 7 days following the infection, 10 fish were collected from each of the appropriate tank at each timepoint randomly. (Ten biological replicates were set up from each timepoint and treatment.) Intestine samples from each fish were collected and frozen in liquid nitrogen immediately and stored at -80 °C until RNA extraction.

### 2.3 Total RNA Extraction

Prior to RNA extraction, individual samples were ground into powder in the presence of liquid nitrogen using a mortar and pestle. Total RNA was extracted according to the instructions of the RNeasy Plus Universal Mini Kit (Qiagen, China). Then the quantity and quality of RNA were determined by the BioTek microplate reader and denaturing agarose gel electrophoresis.

### 2.4 Library Preparation and Sequencing

The NEB Next^®^ Ultra™ RNA Library Prep Kit (NEB, United States) was used to create cDNA libraries. First Strand Synthesis Reaction Buffer, random primers, and reverse transcriptase (Invitrogen, China) were used to synthesize the first-strand cDNA. The second-strand cDNA was synthesized and purified, followed by end repair and adapter ligation. Qubit 2.0 was used to quantify the chosen products enriched by PCR amplification. Sequencing was performed using the Illumina HiSeq 4000, and the sequence data were obtained by Illumina Pipeline Software v1.6 (Biomarker Technologies Inc., Beijing, China).

### 2.5 Bioinformatics Analysis

#### 2.5.1 Quality Control of Sequencing Data

Through trimming the adapter-only sequences, empty reads, poly-N stretches (>10% total N), low-quality reads, and uncertain nucleotide, we obtained clean data from raw data using the fastq filter, and the clean data were then assembled by SOAP *de novo* program. In addition, the Q10, Q20, Q30, GC content, and sequence duplication level of the clean data were simultaneously calculated. All following analyses were based on the high-quality clean data.

#### 2.5.2. Read Mapping to the Reference Genome

The Nile tilapia reference genome and gene model annotation file were downloaded from the NCBI (https://www.ncbi.nlm.nih.gov/genome/197), Kyoto Encyclopedia of Genes and Genomes. The index of the reference genome was built using Bowtie v2.2.3, and paired-end clean reads were aligned to the reference genome using TopHat v2.0.12.

#### 2.5.3 Differential Expression Analysis and Enrichment Analysis

The read numbers mapped to each gene was counted by HTSeq v0.6.1. Fragments per kilobase of transcript per millions base pairs sequenced (FPKM) was used to measure the expression levels of each sample’s transcripts or genes. Differentially expressed genes (DEGs) between each group were explored using DEseq, and we used the Benjamini–Hochberg procedure to assess the statistical significance. Genes were considered to be differentially expressed when their |fold change| ≥ 2 and the false discovery rate (FDR) < 0.01. All the DEGs were further annotated by querying against the GO and KEGG databases. The results were obtained using the GO-seq and KOBAS (2.0), respectively.

### 2.6 Experimental Validation Using qPCR

To validate the results obtained by RNA-seq, eight genes were used for quantitative real-time PCR (RT-qPCR) analysis. Primer 5.0 was used to design the gene specific primers based on the contig sequence (Table 1). The RNA samples used for RT-qPCR amplification were the same as those used to construct the RNA-seq library mentioned earlier. The RT-qPCRs were performed on the CFX96 real-time PCR detection system (BioRad) with SYBR Premix Ex Taq™ (TaKaRa). PCR cycling’s initial degeneration was at 95°C (30 s), followed by 40 cycles of degeneration at 94°C (5s) and appropriate annealing/expansion temperature (60°C, 30 s), and additional temperature-increasing step of 65–95°C was used to generate the melting curve, in response to the fluorescence intensity of three biological replicates of each gene products, with a threshold cycle (Ct) value, the non-template amplification was run as control for each experiment, then the relative quantitative were translated into fold change.

**TABLE 1:** Primers used in this study.

Primer name	Forward primer (5′-3′)	Reverse primer (5′-3′)
*IgM-VH*	GCA​CAG​CAG​AGC​AAA​AAT​GAC	TGG​TTA​GGT​CCG​ACT​CCG​A
*IgG-Fc*	CGC​TGT​GCG​GGT​CAC​TTA​CT	CCA​GGA​GCC​GAC​AAC​TAC​AGA
*IL-1β*	TGA​CGA​CAA​GCC​AAC​CCT​C	TCT​CCT​GAC​ACA​CTT​CCA​CCA
*PSMB8*	ATG​GAC​AGC​GGT​TAC​AAG​GAG	CGT​CCT​GCT​TAC​ACA​CCT​TTA​TC
*ATP-γ*	GGA​GAC​CAA​CTA​CAG​GGA​GAG​G	GTG​ATG​CTG​CTG​ATG​ACG​CT
*E3-TRIM39*	CCT​CAA​CCT​TCA​CGA​AAT​CCC	CCT​GTT​CTT​CCC​TGA​CAT​CTC​C
*CXCL 10*	GCC​GTG​AAA​AGA​CTC​GTG​ACT	GAA​GGT​CTG​ATG​AGT​TTG​TCG​TC
*CCL 19*	CTCGTCGCAAACTACCG	AGCGTTTGGGCTTGTAG
*β-actin*	AAC​AAC​CAC​ACA​CCA​CAC​ATT​TC	TGT​CTC​CTT​CAT​CGT​TCC​AGT​TT

### 2.7 Statistical Analysis

The statistical difference of results were analyzed by the independent sample *t* test in the SPSS statistics tool, then the data were expressed as mean ± standard deviation, and compared the change in relative gene expression between RT-qPCR and RNA-seq.

## 3 Results

### 3.1 Identification of Clean Reads and Gene Annotation

A total of 5,870 million clean data were obtained from each sample. Clean data with similar Q30 base percentage more than 92.05% were obtained from all groups. Moreover, 61.31–87.63% from each group were matched to reference genomic sequence, respectively (Supplementary Tables S1,S2).

### 3.2 Analysis of Differentially Expressed Genes

A total of 18,972 genes from four groups at each timepoint were identified with clear annotations in intestine samples. Among them, a total of 6,185 differentially expressed genes were detected, and 3,876 and 2,309 genes were significantly upregulated and downregulated, respectively (Supplementary Table S3).

**FIGURE 1:**
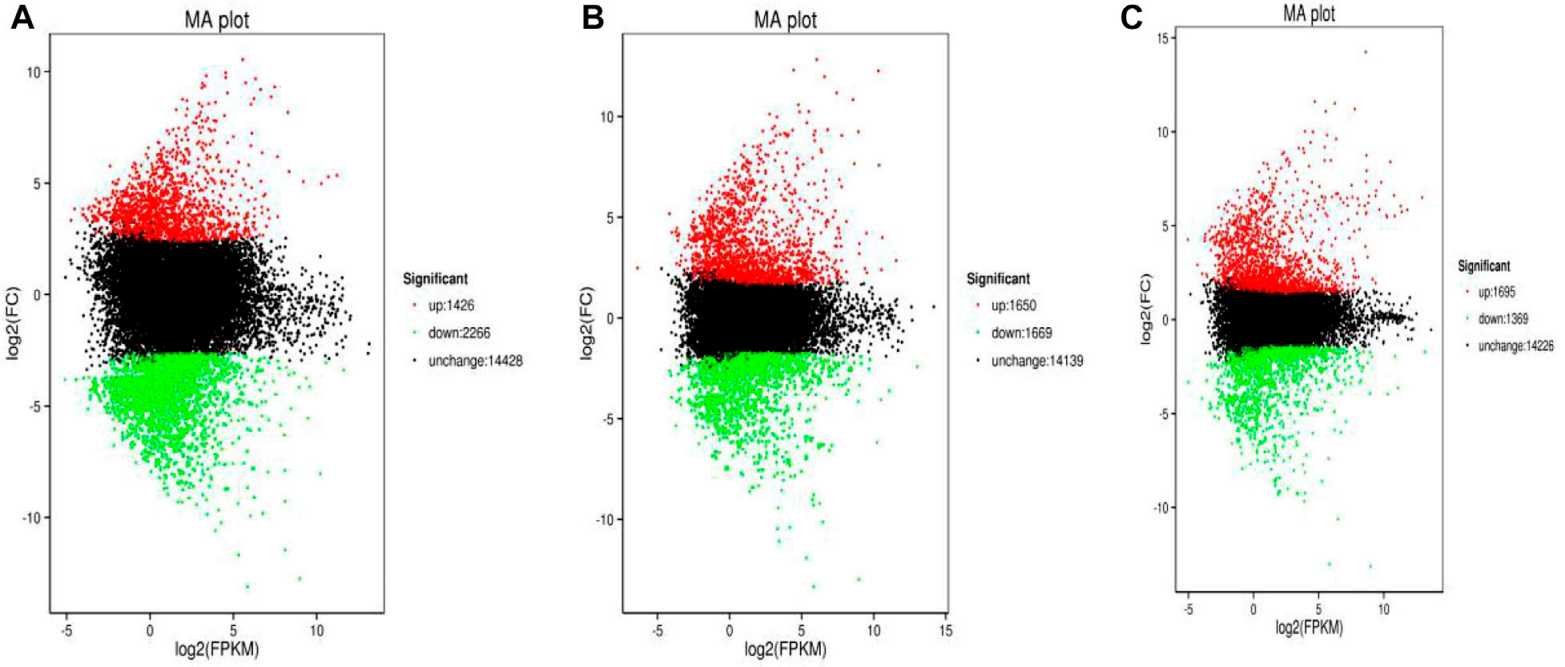
Volcano plot of Nile tilapia DEGs identified from the *Streptococcus* challenge. AG group 24 h vs control was shown in **(A)**, IG group 24 h vs control was shown in **(B)**, while MG group 24 h vs control was displayed in **(C)**. The log2(FPKM) value represented the mean expression level of each gene, and each dot represented one gene. The up-regulated genes were shown in red dots, the down-regulated genes were shown in green dots, while genes with no differential expression were shown in black dots.

The minimum numbers of DEGs in the AG, IG, and the MG groups were 2,874 at 6 h, 2,687 at 12 h, and 2,613 at 6 h, respectively. The maximum number of DEGs in all experimental groups appeared at 24 h (3,792, 3,319, and 3,064, respectively) (Figure 1).

**FIGURE 2:**
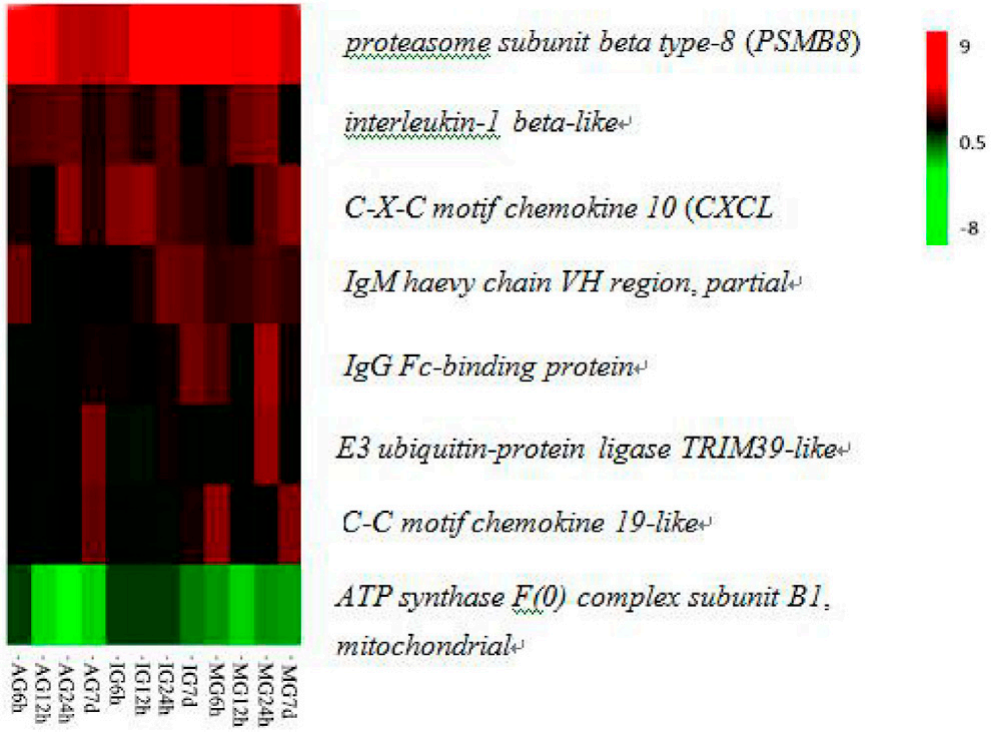
Hierarchical clustering of eight DEGs related to immune responses in the *O. niloticus* intestine.

Through functional annotation and screening, we obtained 136 immunologically DEGs. Among them, we selected eight significant DEGs associated with intestinal inflammation in Nile tilapia including *E3 ubiquitin-protein ligase TRIM39-like*, *C-X-C motif chemokine 10-like(CXCL 10)*, *C-C motif chemokine 19-like*, *interleukin-1 beta-like*, *IgM heavy chain VH region*, *partial*, *IgG Fc-binding protein*, *proteasome subunit beta type-8(PSMB8)*, and *ATP synthase F(0) complex subunit B1*, *mitochondrial* (Figure 2; Table 2).

**TABLE 2:** Differentially expressed immune-related genes from *O. niloticus* following *Streptococcus* challenge.

Gene name	Group	6 h	12 h	24 h		7 d
*Proteasome subunit beta type-8*	AG	**338.8**	257.84		76.5	70.05
	IG	97.45	220.06		**346.31**	**107.74**
	MG	**469.8**	269.28		**352.95**	**111.55**
*ATP synthase F(0) complex subunit B1, mitochondrial*	AG	0.52	**0.02**		**0.01**	**0.02**
	IG	0.55	0.59		0.55	**0.14**
	MG	**0.08**	**0.02**		**0.08**	**0.06**
*E3 ubiquitin-protein ligase TRIM39-like*	AG	1.85	1.69		1.93	**2.07**
	IG	1.42	1.4		**3.2**	1.73
	MG	1.61	1.79		**34.64**	**1.8**
*C-X-C motif chemokine 10*	AG	3.69	1.83		**32.52**	4.81
	IG	**26.45**	**36.02**		9.74	5.57
	MG	3.96	1.83		11.54	**30.11**
*Interleukin-1 beta-like*	AG	9.21	**12.12**		**14.35**	4.55
	IG	10.66	4.49		11.4	**0.76**
	MG	**5.58**	**16.61**		**21.88**	**0.83**
*C-C motif chemokine 19-like*	AG	**15.25**	0.56		0.77	1.45
	IG	1.23	3.81		**17.25**	**14.73**
	MG	5.71	**6.67**		**10.68**	7.12
*IgM heavy chain VH region, partial*	AG	0.67	0.61		0.98	**15.16**
	IG	0.58	0.48		**0.35**	3.53
	MG	**0.96**	0.77		0.83	**21.68**
*IgG Fc-binding protein*	AG	0.48	0.45		**15.74**	3.03
	IG	0.65	0.47		2.9	**17.75**
	MG	**1.59**	**0.98**		**32.02**	2.74

Bold values indicate significant fold change relative to control (*p*-value ≤ 0.05).

### 3.3 Analysis of Gene Ontology Enrichment and KEGG.

**FIGURE 3:**
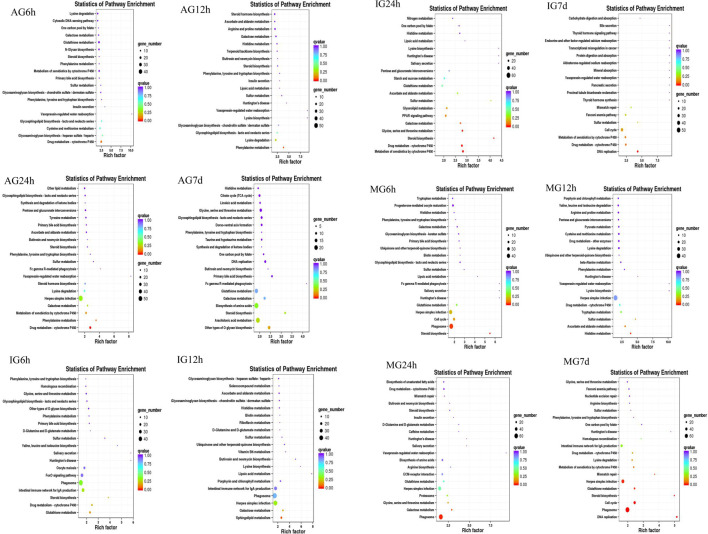
Nile tilapia DEGs in KEGG classification after *Streptococcus* infection.

The differentially expressed genes were annotated for gene ontology by Blast2GO; the gene ontology (GO) terms of different groups were classified to biological process, cellular component, and molecular function. KEGG was used to investigate gene functions. After annotation, 6,185 DEGs were grounded into 100 obviously enriched pathways. The most enriched pathways of each treatment group are shown in Figure 3. The largest enriched pathway was the cytokine–cytokine receptor interaction. In addition, many immune-related pathways also exhibited DEGs enrichment, such as phagosome, calcium signaling pathway, CAMs, proteasome, and oxidative phosphorylation. Some enriched immune-related DEGs in these pathways are shown in Supplementary Table S4.

Among the eight DEGs with significance, *C-X-C motif chemokine 10-like*, *C-C motif chemokine 19-like*, *and interleukin-1 beta-like* belong to the cytokine–cytokine receptor interaction pathway, *IgM heavy chain VH region*, *partial*, *and IgG Fc-binding protein* belonged to the calcium signaling pathway, *E3 ubiquitin-protein ligase TRIM39-like and proteasome subunit beta type-8 (PSMB8)* belonged to the proteasome pathway, and *ATP synthase F(0) complex subunit B1* belonged to the oxidative phosphorylation pathway. These four pathways were related to intestinal inflammation in Nile tilapia (Li et al., 2020). Some key immune-related DEGs were identified from key immune pathways, including the chemokines, immunoglobulin, and ubiquitin proteasome.

### 3.4 Validation of DEGs by RT-qPCR

**FIGURE 4:**
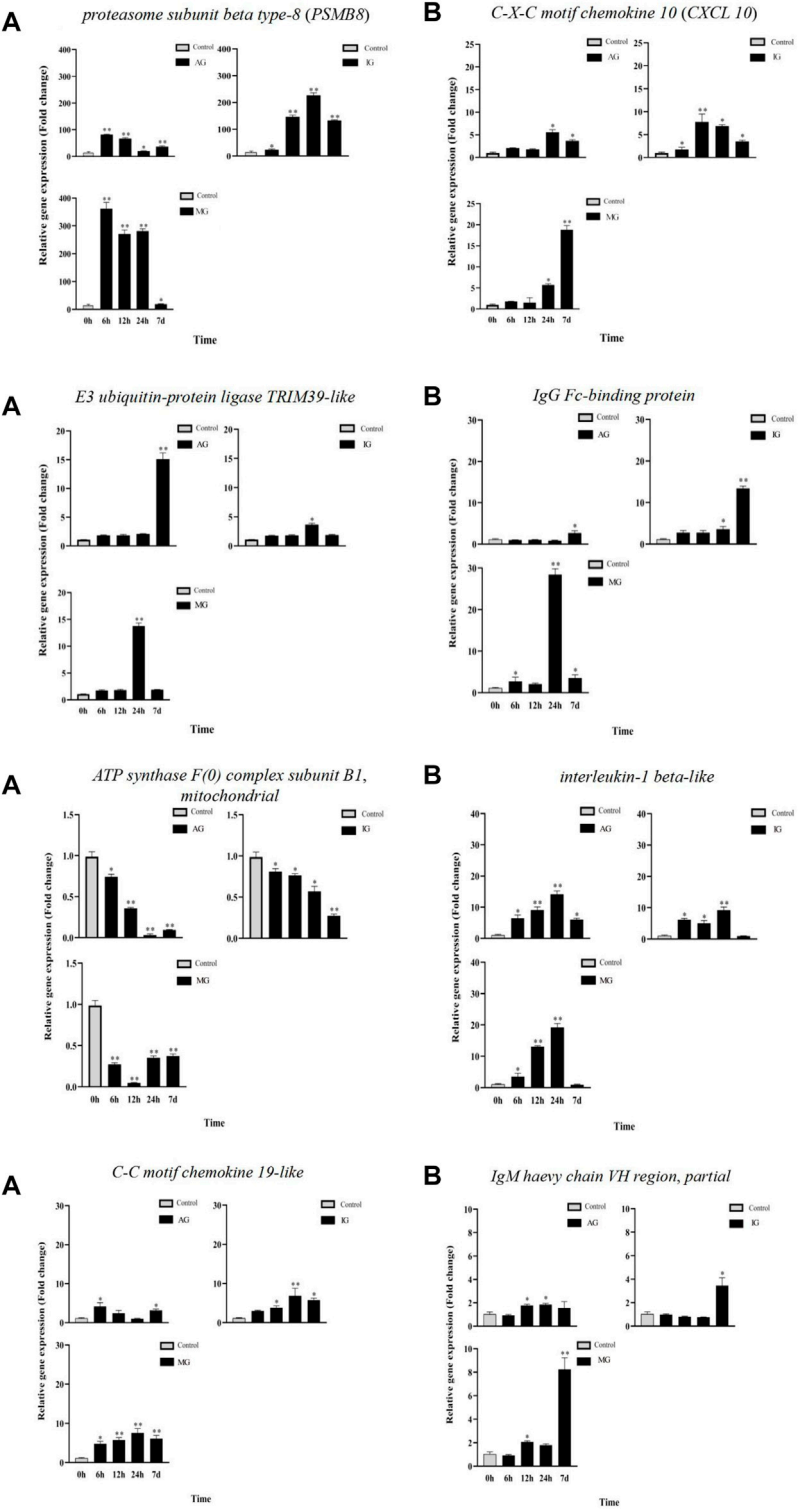
Gene expression analysis from RT-qPCR of Nile tilapia infected with *Streptococcus*. PSMB8, E3 ubiquitin-protein ligase TRIM39-like, ATP synthase F(0) complex subunit B1, mitochondrial and C-C motif chemokine 19-like gene expression analysisi were shown in **(A)**. CXCL 10 , IgG Fc-binding protein, interleukin-1 beta-like and IgM haevy chain VH region, partial gene expression analysis were shown in **(B)**.

To validate the DEGs identified by RNA-seq, eight differentially expressed genes associated with Nile tilapia intestinal inflammation were selected for RT-qPCR confirmation (Figure 4). Melting curve analysis revealed a single product for all tested genes, ensuring the primer specificity. The relative fold changes from RT-qPCR were compared with the RNA-seq expression analysis results. As shown in Supplementary Table S5, the RT-qPCR results revealed that these genes had the same upregulation or downregulation trend with the RNA-seq analysis, indicating the accuracy and reliability of RNA-seq expression analysis.

## 4 Discussion

The intestine is an important immune organ of fish, and a variety of immune cells are distributed in the epithelial layer and mucosa of the fish intestine (Salinas, 2015). Due to the significant role it plays in immune-related disorders, the intestine was chosen for RNA-seq analysis in this study. In order to understand the acute and chronic effects of *Streptococcus* on tilapia, fish samples were collected at 6, 12, 24 h, and 7 days, and 6,185 DEGs were identified from the intestine in tilapia, including 3,876 significantly upregulated genes and 2,309 significantly downregulated genes. Immune-related DEGs in all of the three experimental groups reached the maximum number at 24 h. Previous studies have shown that a certain concentration of bacteria had a stimulating effect on fish (Kacha et al., 2009).

According to the result of functional enrichment analyses, six immune-related pathways were highlighted, including four pathways associated with inflammatory responses (the calcium signaling pathway, the cytokine–cytokine receptor interaction pathway, the proteasome pathway, and the oxidative phosphorylation pathway). These results suggested that single infection or coinfection of *Streptococcus* induced an inflammatory response in the intestines of tilapia.

The intestine is one of the gut-associated lymphoid organs in the body, which can generate great amounts of IgT antibodies, serving as the first line of defense against microbial invasion (Piazzon et al., 2016). It has been reported that the immune-related genes in the calcium signaling pathway were induced in *Paralichthys olivaceus* with the infection of *Edwardsiella tarda* (Xiu et al., 2019). The results of this study revealed the significant enrichment of genes in the calcium signaling pathways, which suggested that this pathway played an important role against the invasion by *Streptococcus*. As part of this pathway, the *IgM heavy chain VH region* and *IgG-Fc binding protein* were significantly upregulated. In addition, the expression of these two genes in the coinfection group was significantly higher than that in the single infection group. *IgG-Fc binding protein* can not only bind to immunoglobulins but also stimulate the proliferation and differentiation of B lymphocytes (Fillatreau et al., 2013). After *Streptococcus* infection, *IgG-Fc binding protein* was significantly upregulated in the intestine within 7 days, implying that *IgG-Fc binding protein*, as an immunoglobulin binding factor, participated in the immune response to bacterial challenge. This result suggested that *Streptococcus* can induce the proliferation and activation of B lymphocytes. This effect was more pronounced in the coinfection group. The IgM encoded by the *IgM heavy chain VH region* is one of the most abundant immunoglobulin molecules in bony fish (Akula and Hellman, 2017). IgM can combine with antigen molecules to form antigen–antibody immune complexes and then be engulfed by macrophages (Gallily et al., 1982). Previous results showed that alginic acid vaccination results in a significant increase in the number of IgM in *Oncorhynchus mykiss* (Gioacchini et al., 2008). In this study, the expression of IgM was significantly upregulated within 7 days in the intestine, suggesting that the *IgM VH region* played an important role in the clearance of *Streptococcus* in the intestine of tilapia. As expected, the expression of the *IgM VH region* in the coinfection group was higher than those in the single infection group, illustrating that coinfection of *S. agalactiae* and *S. iniae* has a more significant stimulating effect on the immune system of tilapia.

The cytokine–cytokine receptor interaction pathway, including soluble extracellular proteins and glycoproteins, played a bridge role between the immune signal and the immune effect (Cui and Ma, 2020). It has been reported that the expression of IL-1β and other pro-inflammatory factors in the cytokine–cytokine receptor interaction pathway was upregulated in the intestine of *Ctenopharyngodon idella* following *Aeromonas hydrophila* infection (Bai et al., 2018). Three significantly differentially expressed genes in the cytokine–cytokine receptor interaction pathway were altered after infection with *S. agalactiae* and *S. iniae*. They were involved in the inflammatory response including *CXCL 10*, *C-C motif chemokine 19-like*, and *interleukin-1 beta-like*. As members of the chemokine family, *CXCL 10* and *C-C motif chemokine* play an important role in the inflammatory response. They mediated and attracted cytokines to the inflammation site to exert immune effects not only in humans but also in teleost fish (Hasni et al., 2017; Ting et al., 2018; Kim et al., 2019). In this study, the expression of *CXCL 10* and *C-C motif chemokine 19-like* were both increased significantly within 24 h in the intestine and then decreased at 7 days. It has reported that *CXCL* was significantly upregulated in the spleen and kidney of tilapia infected by *S. agalactiae* after 6 h (Zhang et al., 2013). Combined our results, we supposed that *CXCL* played an important defensive role in different tissues of tilapia against *Streptococcus* at the early stage. Studies have shown that *CXCL* exerted significant roles in regulating immune cells migration and activation such as B lymphocytes and macrophages (Schwenteit et al., 2013; Mu et al., 2019). This result indicated that after being infected by *Streptococcus*, macrophages and B lymphocytes reached the inflammation area under the mediation and activation of *CXCL 10* and *C-C chemokine 19* in the intestine. *Interleukin 1β* can activate the proliferation and differentiation of immune cells. In this study, the expression of *IL-1β* was upregulated significantly within 24 h in the intestine and then decreased between 24 h and 7 days. Similar to our results, the expression of *IL-1β* significantly increased at 12 h and then decreased between 12 and 24 h following a bacterial infection in *Piaractus mesopotamicus* (Carriero et al., 2020). Our results suggested that after streptococcal infection, B cells and other immune cells were activated by *IL-1β* to protect the organisms from pathogenic stress, exerting a role in modulating the cellular immune response. It is worth mentioning that the expression of these three DEGs in the cytokine–cytokine receptor interaction pathway was significantly higher in the coinfection group than in the single infection group, suggesting that coinfection of *S. agalactiae* and *S. iniae* has a more significant effect on the interaction between immune cells in the intestine.

The *ubiquitin-proteasome* in the proteasome pathway played a significant role in mediating the degradation of intracellular proteins, presenting antigens, and mediating inflammation (Zheng et al., 2020). In the present study, two genes in this pathway were altered after infection with *Streptococcus*, including *ubiquitin-protein ligase TRIM39-like*, and *proteasome subunit beta type-8 (PSMB8).* They were involved in the formation of the ubiquitin proteasome system. In *Paralichthys olivaceus*, the expression of *ubiquitin proteasome* was significantly increased in some immune organs, including gill, heart, muscle, brain, and especially intestine (Liu et al., 2020). The immune catalytic subunit β5i in the ubiquitin-proteasome system was encoded by *PSMB8*, which assisted the activation of antigen-presenting cells to a certain extent (Takezaki et al., 2002). The E3 ubiquitin ligase encoded by *E3 ubiquitin-protein ligase TRIM39-like* catalyzed the ligation of ubiquitin factors to form ubiquitin bodies (Huang et al., 2017). According to our results, the maximum expression of *PSMB8* occurred at 12 h post-injection and then began to decrease from 24 h onward. In the three experimental groups, the expression of *E3 ubiquitin-protein ligase TRIM39-like* also upregulated. In addition, in the coinfection group, the relative expression of PSMD8 was more than 250 times higher than that of the single infection group. Our results revealed that more *ubiquitin proteasomes* were formed in the tilapia to participate in the immune response against the coinfection of *S. agalactiae* and *S. iniae*.

The oxidative phosphorylation pathway played an important role in the synthesis of ATP and the release of reactive oxygen species (ROS) (Nolfi-Donegan et al., 2020). As an active molecule, ROS oxidized metabolic wastes and mediated and regulated a variety of signal transduction pathways (Gauron et al., 2013). In this study, the oxidative phosphorylation pathway was significantly enriched. Furthermore, the expression of *ATP synthase F(0) complex subunit B1* significantly decreased. The core catalytic subunit γ chain subunit B of ATPase encoded by ATP synthase F (0) complex subunit B1 can catalyze the synthesis of ATP (Schredelseker and Pelster, 2004). Taken all these information together, we speculated that *Streptococcus* inhibited the synthesis of ATP in the intestine. This inhibition trend was more obvious in the coinfection group.

**FIGURE 5:**
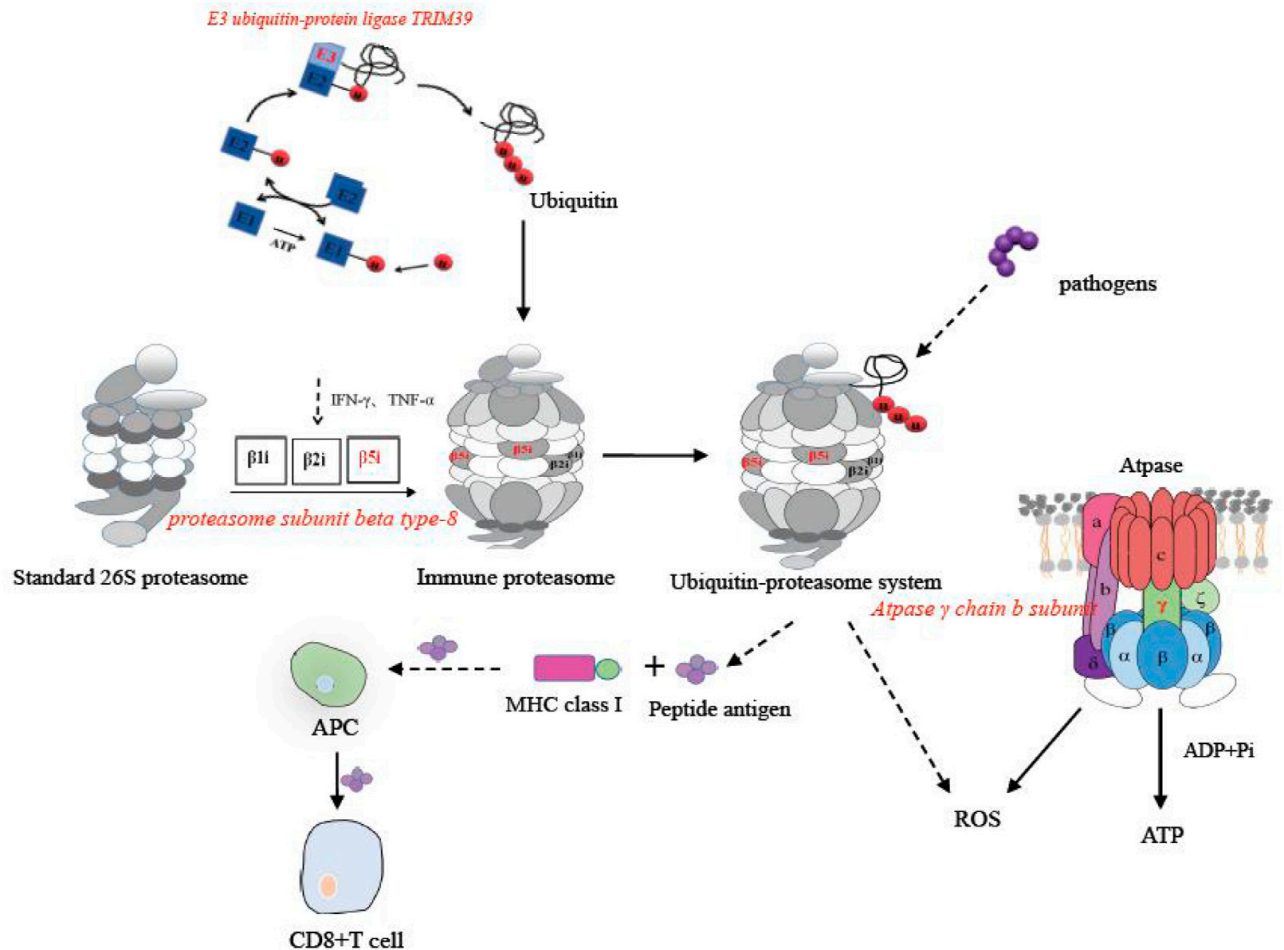
Immune pathways of *PSMB8*, *E3 ubiquitin-protein ligase TRIM39-like*, and *ATP synthase F(0*) *complex subunit B1* in the *O. niloticus* intestine.

**FIGURE 6:**
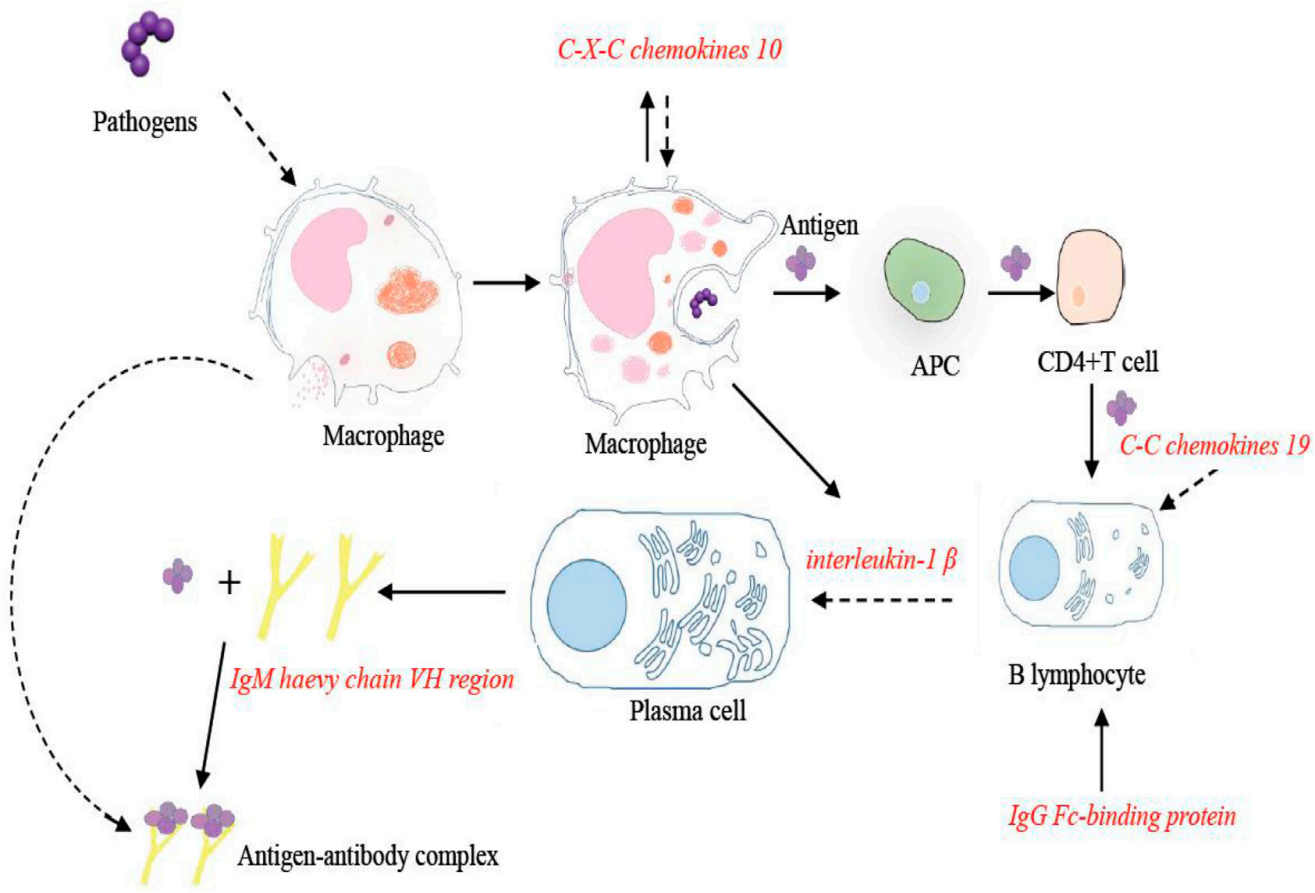
Immune pathways of *CXCL 10*, *C-C motif chemokine 19-like*, *interleukin-1 beta-lik*e, *IgM heavy chain VH region, partial*, and *IgG Fc-binding protein* in the *O. niloticus* intestine.

Immune pathways of eight selected immune DEGs in the *O. niloticus* intestine are shown in Figures 5,6.

## 5 Conclusion

The results in this study demonstrated two potential immune pathways in the intestine of tilapia infected by *S. agalactiae* or *S. iniae*. These two immune pathways medicate inflammatory responses mainly by activating the expression of *E3 ubiquitin-protein ligase TRIM39-like*, *CXCL 10*, *C-C motif chemokine 19-like*, *interleukin-1 beta-like*, *IgM heavy chain VH region*, *partial*, *IgG Fc-binding protein*, *PSMB8*, and *ATP synthase F(0) complex subunit B1, mitochondrial*. In addition, the expression of these eight genes in the MG group was significantly higher than that in the AG and IG groups, which suggested that the coinfection of *S. agalactiae* and *S. iniae* is more significant than the single infection of each of the two pathogens on the stimulation of inflammatory response in tilapia intestines at equivalent concentrations of bacteria. Our study provided a theoretical basis for analyzing the complex molecular mechanism of tilapia *Streptococcus* infection response and a reference for the further research of other fish against streptococcal disease.

## Data Availability

The original contributions presented in the study are publicly available. This data can be found here: National Center for Biotechnology Information (NCBI) BioProject database under accession number PRJNA766813.
