# Supplementary material for: Comparative Transcriptomic Analysis Reveals the Regulated Expression Profiles in Oreochromis niloticus in Response to Coinfection of Streptococcus agalactiae and Streptococcus iniae
Source: Front Genet. 2022 Mar 3;13:782957. doi: 10.3389/fgene.2022.782957 (PMC8927537; doi:10.3389/fgene.2022.782957)
Supplement: Supplementary file 1 [file Table1.docx]

**Supplementary Table 1.** Statistics of clean reads in each group

| Samples name Clean reads Clean bases GC content (%) ≥Q20(%) ≥Q30(%) | | | | | |
| --- | --- | --- | --- | --- | --- |
| CG0h  AG6h  AG12h  AG24h  AG7d  IG6h  IG12h  IG24h  IG7d  MG6h  MG12h  MG24h  MG7d | 24859581  29472156  24928995  20676449  23040419  28339557  30359034  26150007  20966594  28162794  28807741  26122197  23486554 | 7408371186  8806237260  7430803792  6148118254  6870555304  8474679486  9076213534  7808982850  6231326734  8423114166  8599329084  7769524256  7006017288 | 49.05  48.01  44.99  45.23  49.16  47.21  47.84  45.23  48.71  47.19  45.23  49.84  49.47 | 97.72  97.67  97.09  96.58  97.62  97.24  97.53  97.17  97.24  97.18  97.14  97.76  97.65 | 94.04  93.82  92.83  92.05  93.88  92.87  93.55  92.91  93.39  92.74  92.94  94.15  93.88 |

**Supplementary Table 1.** Statistics of reads matched to reference genomic sequence

| Samples name | Total Reads | Mapped Reads | Uniq Mapped Reads | Multiple Map Reads | Reads Map to '+' | Reads Map to '-' |
| --- | --- | --- | --- | --- | --- | --- |
| CG0h | 49,719,162 | 43,447,562 (87.39%) | 41,901,969 (84.28%) | 1,545,593 (3.11%) | 21,590,627 (43.43%) | 21,726,763 (43.70%) |
| AG6h | 58,944,312 | 50,987,672 (86.50%) | 49,361,216 (83.74%) | 1,626,456 (2.76%) | 25,333,317 (42.98%) | 25,539,077 (43.33%) |
| AG12h | 49,857,990 | 40,675,107 (81.58%) | 39,752,909 (79.73%) | 922,198 (1.85%) | 20,183,814 (40.48%) | 20,359,926 (40.84%) |
| AG24h | 41,352,898 | 25,353,277 (61.31%) | 24,885,211 (60.18%) | 468,066 (1.13%) | 12,458,972 (30.13%) | 12,745,101 (30.82%) |
| AG7d | 46,080,838 | 39,140,345 (84.94%) | 38,018,399 (82.50%) | 1,121,946 (2.43%) | 19,450,472 (42.21%) | 19,567,226 (42.46%) |
| IG6h | 56,679,114 | 48,521,441 (85.61%) | 47,129,080 (83.15%) | 1,392,361 (2.46%) | 24,110,915 (42.54%) | 24,265,743 (42.81%) |
| IG12h | 60,718,068 | 53,101,571 (87.46%) | 51,434,813 (84.71%) | 1,666,758 (2.75%) | 26,394,783 (43.47%) | 26,531,428 (43.70%) |
| IG24h | 52,300,014 | 42,709,177 (81.66%) | 41,662,645 (79.66%) | 1,046,532 (2.00%) | 21,185,745 (40.51%) | 21,312,519 (40.75%) |
| IG7d | 41,933,188 | 36,026,558 (85.91%) | 34,746,447 (82.86%) | 1,280,111 (3.05%) | 17,902,212 (42.69%) | 18,047,830 (43.04%) |
| MG6h | 56,325,588 | 48,250,895 (85.66%) | 46,876,267 (83.22%) | 1,374,628 (2.44%) | 23,976,925 (42.57%) | 24,110,579 (42.81%) |
| MG12h | 57,615,482 | 48,617,450 (84.38%) | 47,365,430 (82.21%) | 1,252,020 (2.17%) | 24,143,482 (41.90%) | 24,289,435 (42.16%) |
| MG24h | 52,244,394 | 45,782,768 (87.63%) | 43,803,063 (83.84%) | 1,979,705 (3.79%) | 22,765,371 (43.57%) | 22,868,192 (43.77%) |
| MG7d | 46,973,108 | 40,299,603 (85.79%) | 38,711,292 (82.41%) | 1,588,311 (3.38%) | 20,017,991 (42.62%) | 20,177,782 (42.96%) |

Note：Uniq Mapped Reads represent the percentage of reads that compared to the *O. niloticus* reference genome at the unique loci; Multiple Map Reads represent the percentage of reads that compared to the *O. niloticus* reference genome at the multiple loci;Reads Map to '+' represent the percentage of reads on the positive strand alignment of the *O. niloticus* reference genome to the Clean Reads; Reads Map to '-' represent the percentage of reads on the negative strand alignment of the *O. niloticus* reference genome to the Clean Reads.）

**Supplementary Table 3.** Statistics of DEGs number in each experimental group at four time points

|  | 6h | | 12h | | 24h | | 7d | |
| --- | --- | --- | --- | --- | --- | --- | --- | --- |
|  | up | down | up | down | up | down | up | down |
| AG | 1258 | 1616 | 1298 | 2096 | 1426 | 2266 | 2452 | 1116 |
| IG | 1659 | 1245 | 1415 | 1272 | 1650 | 1669 | 1211 | 1706 |
| MG | 1604 | 1009 | 1183 | 1864 | 1695 | 1369 | 1679 | 1368 |

**Supplementary Table 4.** Some enriched immune-related DEGs under 4 significantly different pathways

| Group | Protein name | Gene ID |
| --- | --- | --- |
| Phagosome |  |  |
| AG group | complement C3 | gene:ENSONIG00000016413 |
|  | complement C1q tumor necrosis factor-related protein 3-like | gene:ENSONIG00000011744 |
|  |  | gene:ENSONIG00000013828 |
|  |  | gene:ENSONIG00000008318 |
|  |  | gene:ENSONIG00000000361 |
|  |  | Oreochromis_niloticus_newGene_31450 |
|  |  | gene:ENSONIG00000008737 |
|  |  | Oreochromis_niloticus_newGene_31446 |
|  | HLA class II histocompatibility antigen | gene:ENSONIG00000019001 |
|  |  | gene:ENSONIG00000006327 |
|  |  | gene:ENSONIG00000006325 |
| IG group | complement C3 | gene:ENSONIG00000016413 |
|  | complement C1q tumor necrosis factor-related protein 3 | gene:ENSONIG00000008535 |
|  |  | gene:ENSONIG00000013828 |
|  |  | gene:ENSONIG00000008737 |
|  |  | gene:ENSONIG00000000361 |
|  | HLA class II histocompatibility antigen | Oreochromis_niloticus_newGene_44441 |
|  |  | gene:ENSONIG00000019001 |
|  |  | gene:ENSONIG00000006327 |
|  |  | gene:ENSONIG00000019943 |
| MG group | complement C3 | Oreochromis_niloticus_newGene_13450 |
|  |  | gene:ENSONIG00000016413 |
|  | complement C1q tumor necrosis factor-related protein 3-like | gene:ENSONIG00000008535 |
|  |  | gene:ENSONIG00000000339 |
|  |  | gene:ENSONIG00000013828 |
|  |  | gene:ENSONIG00000000361 |
|  | HLA class II histocompatibility antigen | gene:ENSONIG00000012499 |
|  |  | gene:ENSONIG00000019943 |
|  |  | gene:ENSONIG00000018290 |
|  |  | Oreochromis_niloticus_newGene_17646 |
|  |  | Oreochromis_niloticus_newGene_46364 |
| Cell adhesion molecules (CAMs) |  |  |
| AG group | HLA class II histocompatibility antigen | gene:ENSONIG00000012499 |
|  |  | gene:ENSONIG00000019943 |
|  |  | gene:ENSONIG00000018290 |
|  |  | Oreochromis_niloticus_newGene_17646 |
|  |  | Oreochromis_niloticus_newGene_46364 |
| IG group | neural cell adhesion molecule 2-like | Oreochromis_niloticus_newGene_33405 |
|  | HLA class II histocompatibility antigen | gene:ENSONIG00000007425 |
|  |  | gene:ENSONIG00000007449 |
|  |  | gene:ENSONIG00000007453 |
|  |  | gene:ENSONIG00000019943 |
|  |  | gene:ENSONIG00000006327 |
|  | neural cell adhesion molecule L1-like isoform X1 | gene:ENSONIG00000002282 |
| MG group | neural cell adhesion molecule 2-like | Oreochromis_niloticus_newGene_33405 |
|  | HLA class II histocompatibility antigen | gene:ENSONIG00000006325 |
|  |  | gene:ENSONIG00000019943 |
|  |  | gene:ENSONIG00000006327 |
| Cytokine-cytokine receptor interaction |  |  |
| AG group | C-X-C motif chemokine 10-like | gene:ENSONIG00000013990 |
|  | C-C motif chemokine 19-like | gene:ENSONIG00000014063 |
|  |  |  |
|  | interleukin-1 beta-like | gene:ENSONIG00000008343 |
|  |  |  |
|  | interleukin-8 | Oreochromis_niloticus_newGene_45404 |
|  | C-type lectin domain family 4 member M-like | gene:ENSONIG00000018314 |
|  |  | gene:ENSONIG00000007092 |
| IG group | C-X-C motif chemokine 10-like | gene:ENSONIG00000013990 |
|  | C-C motif chemokine 19-like | gene:ENSONIG00000014063 |
|  | interleukin-1 beta-like | gene:ENSONIG00000016996 |
|  |  | gene:ENSONIG00000008343 |
|  | interleukin-8 | Oreochromis_niloticus_newGene_45404 |
| MG group | C-X-C motif chemokine 10-like | gene:ENSONIG00000013990 |
|  | C-C motif chemokine 19-like | gene:ENSONIG00000014063 |
|  | interleukin-1 beta-like | gene:ENSONIG00000008343 |
|  |  | Oreochromis_niloticus_newGene_21585 |
|  | interleukin-8 | Oreochromis_niloticus_newGene_45404 |
| calcium signaling pathway |  |  |
| AG group | IgM heavy chain VH region, partial | Oreochromis_niloticus_newGene_38144 |
|  | IgG Fc-binding protein | gene:ENSONIG00000016477 |
|  | HLA class II histocompatibility antigen | gene:ENSONIG00000019001 |
|  |  | gene:ENSONIG00000006327 |
|  | V-set and immunoglobulin domain-containing protein 1-like isoform X1 |  |
|  |  | gene:ENSONIG00000008284 |
|  |  | gene:ENSONIG00000001820 |
| IG group | IgM heavy chain VH region, partial | Oreochromis_niloticus_newGene_38144 |
|  | IgG Fc-binding protein | gene:ENSONIG00000016477 |
|  | HLA class II histocompatibility antigen | gene:ENSONIG00000012437 |
|  |  | gene:ENSONIG00000007453 |
|  | V-set and immunoglobulin domain-containing protein 1-like isoform X1 | Oreochromis_niloticus_newGene_26627 |
|  |  | Oreochromis_niloticus_newGene_17559 |
| MG group | IgM heavy chain VH region, partial | Oreochromis_niloticus_newGene_38144 |
|  | IgG Fc-binding protein | gene:ENSONIG00000016477 |
|  | HLA class II histocompatibility antigen | gene:ENSONIG00000006325 |
|  |  | gene:ENSONIG00000019943 |
|  |  | gene:ENSONIG00000006327 |
|  | V-set and immunoglobulin domain-containing protein 1-like isoform X1 | Oreochromis_niloticus_newGene_38144 |
|  |  | Oreochromis_niloticus_newGene_44965 |

**Supplementary Table 5.** Correlation analysis of RNA-seq and qPCR of 8 immune DEGs

| Sequence | Gene name | correlation coefficient R |
| --- | --- | --- |
| 1 | *E3 ubiquitin-protein ligase TRIM39-like* | 0.85223 |
| 2 | *ATP synthase F(0) complex subunit B1*, *mitochondrial* | 0.91706 |
| 3 | *proteasome subunit beta type-8* | 0.82023 |
| 4 | *C-X-C motif chemokine 10-like* | 0.92084 |
| 5 | *C-C motif chemokine 19-like* | 0.92869 |
| 6 | *IgM haevy chain VH region*, *partial* | 0.93114 |
| 7 | *IgG Fc-binding protein* | 0.91227 |
| 8 | *interleukin-1 beta-like* | 0.97902 |
